# Hysteroscopic and aspiration biopsies in the histologic evaluation of the endometrium, a comparative study

**DOI:** 10.1097/MD.0000000000017183

**Published:** 2019-10-04

**Authors:** Gilberto Massaki Utida, Jaime Kulak

**Affiliations:** a Hospital e Maternidade São José dos Pinhais; b Post-Graduation Program, Federal University of Parana (UFPR), Curitiba, Paraná, Brazil.

**Keywords:** biopsy/methods, endometrial neoplasms, endometrium, hysteroscopy/instrumentation, hysteroscopy/methods

## Abstract

This study aimed to compare the quality of histological endometrial samples collected through Pipelle aspiration and hysteroscopic biopsies to assess the agreement between these 2 biopsies in the histological diagnosis of malignancy and to compare the costs of both biopsies.

This was a cross-sectional study. Forty-five women were biopsied, first using Pipelle and immediately after using hysteroscopy. The material collected was sent for analysis, and hysteroscopy was considered the gold standard. The results were divided into the following 3 categories: normal (atrophic, proliferative, and secretory endometrium); polyps; and malignancies. We report the agreement between Pipelle and hysteroscopy in the diagnosis of malignancy and compare their costs.

The study showed that while analyzing endometrial malignancies, Pipelle sampling had 100% sensitivity and specificity. In the detection of polyps, Pipelle sampling showed 26.1% sensitivity, 88.9% specificity, 75% positive predictive value, 48.5% negative predictive value, and 53.7% accuracy. Agreement with hysteroscopy in the diagnosis of malignancy was 100%. The Pipelle device costs 27 times less than hysteroscopic biopsy for health insurance companies. This cost is 13.7 times lower in the Brazilian Unified Health System.

Endometrial biopsies using the Pipelle have a high accuracy for endometrial cancer and a low accuracy for polyps. We detected 100% agreement between the reports of Pipelle and hysteroscopy with regard to malignancy. Pipelle is the most cost-effective method of endometrial biopsy.

## 1 Introduction

Abnormal uterine bleeding (AUB) occurs in 60% to 70% of women at the end of menarche, during menopause transition, and during menopause.^[1]^ Endometrial cancer is the most common gynecologic tumor in developed countries and the cancer with the seventh-highest incidence in the Brazilian female population.^[2,3]^ Women with AUB and postmenopausal bleeding often require endometrial evaluation with biopsy, with the main purpose of these biopsies being to rule out malignancy.^[4]^ Presently, the gold standard for endometrial biopsy is hysteroscopy, which enables the direct visualization of the uterine cavity and targeted biopsy.^[2]^ Outpatient Pipelle (aspiration) endometrial biopsy has been performed since 1984. It uses a flexible, easy to handle, practical, and low-cost polypropylene cannula, which does not require hospitalization.^[5]^ Soon after its introduction, Pipelle biopsy was quickly assimilated by several countries to evaluate AUB and postmenopausal bleeding, currently being the most frequently used outpatient endometrial biopsy method in countries such as the United States, England, the Netherlands, and New Zealand.^[6–9]^ Pipelle was adopted because of its ease of use and high accuracy, especially in the diagnosis of endometrial cancer.^[2,10–13]^ In the studies published so far, the comparative results of endometrial biopsy have been analyzed at different times, with intervals generally ranging between 60 and 180 days. In this period, endometrial changes may occur and compromise the results. The aim of this study was to compare the quality of endometrial histological samples from Pipelle biopsy with that of samples collected by hysteroscopy (gold standard) to assess the correlation between Pipelle endometrial and hysteroscopic biopsies in the diagnosis of malignancy and to compare the costs of Pipelle and hysteroscopic sampling.

## 2 Methods

This was a cross-sectional study that assessed 2 diagnostic methods applied to the same individual by analyzing the sensitivity and specificity of Pipelle with hysteroscopy as the gold standard. Information on the cost of both procedures was obtained from 2 health insurance companies established in the region where the study was conducted and from the Brazilian Unified Health System (SUS).

Forty-five patients underwent 2 sequential biopsies (Pipelle and hysteroscopy, respectively).

The inclusion criteria for this study were: women aged over 35 years of age with an indication for endometrial biopsy (due to AUB and postmenopausal bleeding) who sought public and private specialized services in 2016 and 2017 in 2 different hospitals. Any patient who did not meet the aforementioned criteria was excluded from the study (Table 2).

The study took place in *Hospital e Maternidade São José—*public hospital and *Hospital Nova Clínica—*private hospital, in the city of São José dos Pinhais, Paraná, Brazil.

Over the course of two years, for the purpose of this study, these women were treated in the hospitals they were admitted to. All procedures regarding the study were performed by G.M.U.

We standardized the transvaginal ultrasound reports using the same ultrasound machine (Toshiba, model 300 Aplio/TUS-A300; Japan) and having the examinations conducted by the same qualified professional in order to measure the thickness of the endometrium.

The Pipelle used is manufactured by CooperSurgical, with the Pipelle trademark.

A Bettocchi hysteroscope from Karl Storz (Germany) was used for this study. The distending medium for the uterine cavity was 0.9% saline. Microscissors and grasping forceps were used for the endometrial biopsy.

The procedure followed a standardized sequence; the Pipelle biopsy was performed first and hysteroscopy with biopsy was conducted immediately after. The vials with the tissue samples were labeled with the tags “endometrial 1” and “endometrial biopsy 2.” The pathologist was kept blind to which method had been utilized for each biopsy.

For statistical analysis, the results of the quantitative variables were described by means and standard deviations or medians and amplitudes. The categorical variables were expressed as frequencies and percentages. The Student *t* test for independent samples or the Mann–Whitney nonparametric test were used for comparing the quantitative variables between 2 groups. Three groups (normal, polyps, and malignancies) were compared using analysis of variance with 1 factor or the Kruskal–Wallis nonparametric test. Normality of the continuous variables was evaluated using the Kolmogorov–Smirnov test. For comparing the categorical variables, the Chi-squared or Fisher exact tests were used. The quality of the microscopic analysis with Pipelle collection was evaluated by estimating sensitivity, specificity, probability of false positives and false negatives, accuracy, and positive and negative predictive values. The result of the microscopic analysis with hysteroscopy collection was considered as the gold standard. The Kappa coefficient was used as measure of agreement between the 2 methods. Statistical significance was set at *P* < .05. Data were analyzed using the SPSS Statistics v. 20.0. software (IBM Corp, Armonk, NY).

The study was approved by the Research Ethics Committee of the Hospital das Clínicas of the Federal University of Paraná (UFPR), under No. 1699216. A written informed consent form was obtained from all patients.

## 3 Results

Of the initially recruited 45 patients, 23 were premenopausal and 22 were postmenopausal. Four patients were excluded from the analyses. There was 1 perforation during hysterometry, 2 cases of insufficient material in Pipelle biopsy, and 1 case of insufficient material in hysteroscopy (without any possible biopsy area) (Table 1).

**Table 1:**
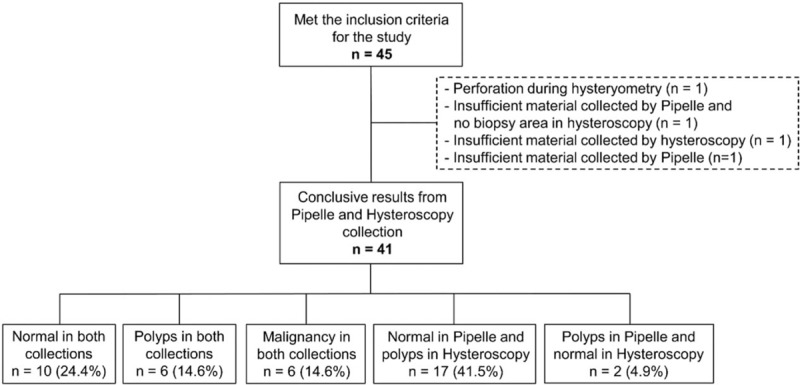
Study Diagram.

**Table 2:**
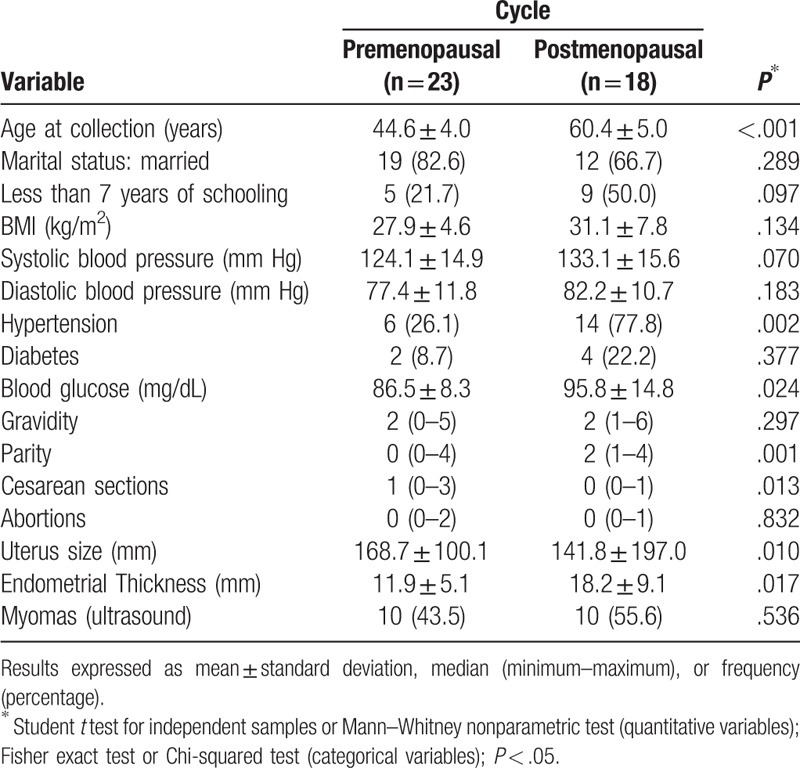
Characteristics of the patients in the study (n = 41).

A complete analysis of the samples was possible in 41 patients. The samples collected using Pipelle enabled the histological identification of 27 patients with normal endometrium, 8 patients with polyps, and 6 patients with malignancies. The samples collected using hysteroscopy enabled the identification of 12 patients with normal endometrium, 23 patients with polyps, and 6 patients with malignancies (Tables 1 and 3). In the detection of polyps, Pipelle sampling showed 26.1% sensitivity, 88.9% specificity, 75% positive predictive value, 48.5% negative predictive value, and 53.7% accuracy (Table 4).

**Table 3:**
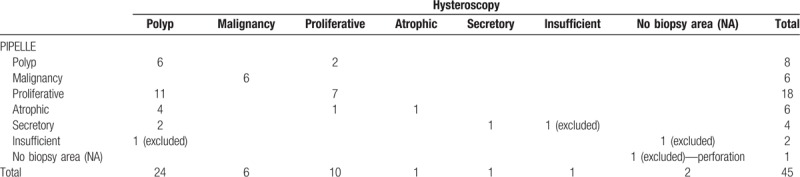
Histological results of biopsy with Pipelle and with hysteroscopy.

**Table 4:**
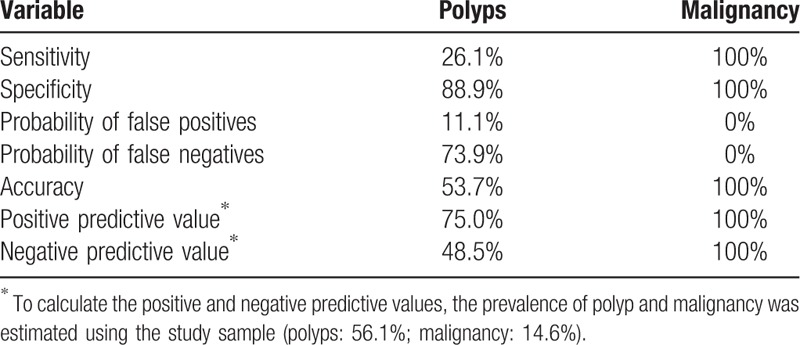
Evaluation of the quality of Pipelle collection in the diagnosis of polyps and malignancy, considering hysteroscopy collection the gold standard (n = 41).

The 6 cases of malignancy were diagnosed using Pipelle and hysteroscopy with 100% agreement, of which 4 were endometrioid adenocarcinomas, 1 was papillary serous carcinoma, and another was leiomyosarcoma (with degeneration, softened, and invading the uterine cavity).

With regard to procedure costs, each Pipelle device used in the study costed R$20. The amounts were obtained from the documents “Rol Hierarquizado Unimed Curitiba” (Unimed Curitiba List) (RHUC) (2016)^[14]^ and “Classificação Brasileira Hierárquica de Procedimentos Médicos” (Brazilian Hierarchical Classification for Medical Procedures) (CBHPM) (2005),^[15]^ used by insurance companies 01 and 02, respectively. For the outpatient endometrial biopsy procedure, with code 31303030 of medical procedures, companies 01 and 02 paid doctors R$48.00 and R$33.60, respectively. Adding the cost of a Pipelle curette to the amount paid by the health insurance company, the total cost of the Pipelle biopsy procedure came to R$53.60 and R$68.00 for companies 01 and 02, respectively (Table 5).

**Table 5:**
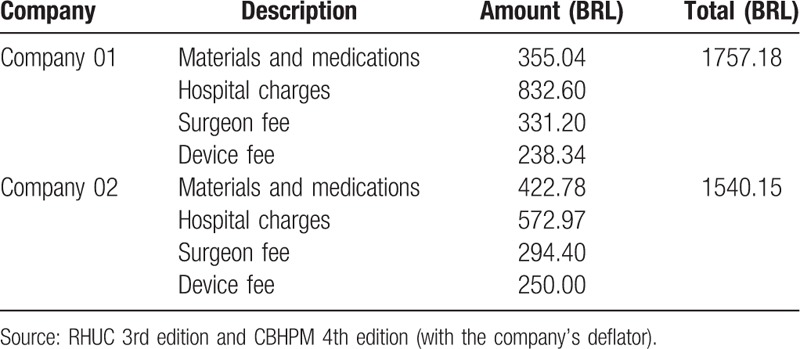
Cost of the hysteroscopy with biopsy procedure, company 01, and company 02.

When comparing the cost of a Pipelle biopsy with that of a hysteroscopic biopsy, the latter costs health insurance companies 27 times more.

The SUS does not have a table of medical fees for the outpatient endometrial biopsy procedure. Hospitals are paid R$198.81 for every patient hospitalized for AUB, plus R$76.50 to perform the hysteroscopy, which totals to R$275.31 (Table 6).^[16]^ Thus, a Pipelle biopsy costs 13,7 times less than a hysteroscopic biopsy.

**Table 6:**
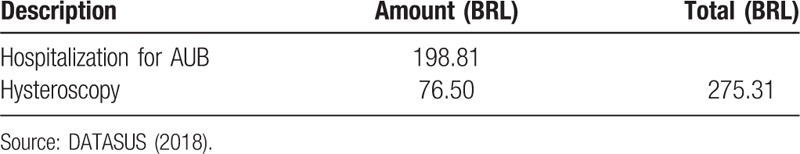
Cost of the hysteroscopy with biopsy procedure, Brazilian Unified Health System.

## 4 Discussion

There are no recommended protocols for endometrial cancer screening.^[17]^ Appropriate guidance and thorough evaluation of patients with AUB, especially those with postmenopausal bleeding, are the primary strategies to aid in the early detection of endometrial malignancies.^[13]^ In addition, early outpatient endometrial evaluation, with its low cost and ease of use, is crucial. For this reason, Pipelle is widely used in the diagnosis of endometrial cancers in countries such as the United States, England, and New Zealand.^[6–9]^

Our study found a high accuracy in the diagnosis of endometrial malignancies, a finding that is corroborated by data from the literature reporting high accuracy for malignancy when sufficient material is collected.^[11,12]^

In Brazil, patients with AUB or postmenopausal bleeding are faced with long waiting times for medical visits, preoperative examinations, and hospital beds for uterine curettage in centers of the SUS, which is a burden on the health care system and delays diagnosis and treatment. Patients with private health insurances undergo endometrial biopsies earlier, usually via hysteroscopy. However, this evaluation method is expensive. Although it failed to show good accuracy for focal endometrial lesions such as polyps, Pipelle was shown to have a high accuracy for endometrial cancer and can be used as early as in the first visit of a patient presenting AUB or postmenopausal bleeding. The International Federation of Gynecology and Obstetrics recommends that patients with AUB and risk factors for endometrial cancer undergo outpatient endometrial biopsy at the beginning of the investigation.^[18]^ The American College of Obstetricians and Gynecologists recommends that patients with postmenopausal bleeding undergo Pipelle biopsy at the beginning of the investigation.^[13]^ Reports of the Brazilian Federation of Gynecology and Obstetrics Associations (FEBRASGO) indicate that Pipelle is the most frequently used outpatient endometrial biopsy method.^[19]^

Optimizing endometrial biopsies aiming at speed, low cost, and especially the exclusion of malignancy requires reflection on the strengths and weaknesses of both methods described herein. Although hysteroscopy remains the gold standard in endometrial evaluation, Pipelle was shown to be very useful and could be more widely used in our country.

## Author contributions

**Conceptualization:** Gilberto Massaki Utida, Jaime Kulak Jr.

**Investigation:** Gilberto Massaki Utida.

**Methodology:** Gilberto Massaki Utida, Jaime Kulak Jr.

**Resources:** Gilberto Massaki Utida.

**Supervision:** Jaime Kulak Jr.

**Writing – review & editing:** Gilberto Massaki Utida.
